# The Impact of Metabolic Bariatric Surgery on Inflammatory Bowel Disease Risk and Outcomes in Adults With Obesity: a Propensity-matched, Nationwide, Analysis

**DOI:** 10.1007/s11695-026-08714-1

**Published:** 2026-05-05

**Authors:** Antoinette Pusateri, Yevgeniya Gokun, Kenneth Allen, ChienWei Chang, Adeeti Chiplunker, Madalina Butnariu, Hisham Hussan

**Affiliations:** 1 Division of Gastroenterology, Hepatology & Nutrition, Department of Internal Medicine, The Ohio State University Wexner Medical Center, Columbus, United States; 2 Secondary Data Core, Center for Biostatistics, The Ohio State University College of Medicine, The Ohio State University, Columbus, United States; 3 Gastroenterology & Hepatology, Department of Internal Medicine, University of California, Davis, Davis, United States

**Keywords:** Metabolic bariatric surgery, De novo IBD

## Abstract

**Introduction:**

The risk of de novo inflammatory bowel disease (IBD) after metabolic bariatric surgery (MBS) has been described, but the timing and severity of de novo IBD is unclear.

**Methods:**

Using MarketScan Databases, patients with severe obesity undergoing Roux-en-Y-gastric bypass (RYGB) and vertical sleeve gastrectomy (VSG) were propensity-matched with patients with severe obesity without MBS (controls). Adjusted hazard ratio (aHR) assessed ulcerative colitis (UC) or Crohn’s disease (CD) hazard <3 or ≥3 years from surgery or severe obesity. IBD severity was assessed using healthcare utilization–based proxies including medication exposure, IBD-related hospitalizations, and surgical interventions.

**Results:**

The cohort included 100,832 adults with MBS versus 376,855 controls (76.0% females, median age of 44 years). The incidence of IBD was higher in MBS cohort versus controls (61.2 vs. 44.4 per 100,000 adults/year). Within 3 years, patients with MBS had a 24% lower risk of de novo IBD versus controls (aHR: 0.76, 95% CI: 0.60-0.95). When stratified by surgery and IBD type, VSG had reduction in CD risk (aHR=0.46, 95% CI:0.22-0.96), while RYGB had reduction in UC risk (aHR=0.22, 95% CI:0.06-0.75). After 3 years, patients with MBS, particularly VSG, had greater than 2-fold increased risk of IBD (aHR=2.28, 95%CI: 1.02-5.06). Markers of treatment intensity and healthcare utilization did not significantly differ between groups overall; however, a higher proportion of UC patients in the MBS cohort underwent colectomy, though absolute event numbers were small. (8.70% vs 1.57%, p=0.03).

**Conclusion:**

While MBS may lower IBD risk initially, IBD risk increases after 3 years, especially UC after VSG, and may be more severe as indicated by the higher proportion of colectomies for patients in the MBS cohort versus controls. Findings regarding treatment intensity should be interpreted cautiously, as objective measures of disease activity were not available in claims data.

**Supplementary Information:**

The online version contains supplementary material available at 10.1007/s11695-026-08714-1.

## Key Points

• The timeline and severity of de novo IBD after MBS is unclear.

• This study found the risk of IBD increases 3 years after MBS.

• Patients with MBS and UC had a higher risk of colectomy.

• This study could help stratify those patients undergoing MBS at risk for IBD.

**Supplementary Information:**

The online version contains supplementary material available at 10.1007/s11695-026-08714-1.

## Introduction

Obesity is a rising healthcare problem in the United States with 50% of adults being projected to have obesity in 2030 [1]. The relationship between obesity and inflammatory bowel diseases (IBD) has been analyzed, as the incidence of IBD has also risen [2–4]. A recent meta-analysis identifies a lower risk of IBD, specifically ulcerative colitis (UC), in adults with obesity compared to normal-weight adults [5]. For adults with established IBD, some studies report a milder disease in adults with obesity when compared to normal-weight adults [6–11], while others observe a more severe disease [12–16]. Like colorectal cancer, IBD can lead to weight loss which can confound the relation with obesity as a risk factor [17]. Examining the impact of weight loss therapies on IBD risk and outcomes may provide deeper insights.

Despite innovations in weight loss approaches, metabolic bariatric surgery (MBS) remains the most effective weight loss method in patients with severe obesity [18–21]. In response to the rising rates of obesity, there has been a surge in use of MBS with more than a quarter million surgeries performed per year in the U.S. alone [22–24]. However, in recent years, the diagnosis of de novo IBD after MBS has been reported [25–33]. While a large health claims database study showed a risk reduction in IBD predominantly in individuals able to achieve normalization of BMI within 1 year of the surgery [34], multi-center studies have shown an increased risk in all IBD [35], CD [36] and UC [37] more than 4 years after MBS. The mechanisms of incident IBD after MBS are unclear but include alterations in the microbiome, malabsorption and dietary changes [32, 37–56]. However, these studies linking MBS to IBD risk had limitations including sample size, design, outcomes measures which did not assess IBD outcomes.

In this study, a national claims database was used to analyze the incidence of newly diagnosed IBD in patients with severe obesity undergoing MBS compared to matched controls. The hypothesis was that patients with severe obesity who undergo MBS will have the highest incidence of de novo IBD, but a less severe disease course as indicated by IBD medications, surgeries and hospitalizations when compared to control patients with severe obesity but who did not undergo MBS. Compared to other studies, we used robust diagnostic criteria for IBD and stringent exclusion criteria to reduce potential bias.

## Methods

### The MarketScan Database

This was a retrospective cohort study using the 2012–2021 IBM^®^ MarketScan Research Databases, which contain proprietary de-identified claims data for privately and publicly insured people in the United States. The MarketScan consists of two core claims databases: commercial and Medicare supplement, providing the de-identified healthcare data from over 273 million patients. The MarketScan Research Databases meet the criteria for a limited-use data set and contain none of the data elements prohibited by Health Insurance Portability and Accountability Act of 1996 (HIPAA), thus data is considered deidentified and this project was exempt from Institutional Review Board oversight [57, 58].

### The Cohort

The MarketScan database was queried using International Classification of Diseases, Ninth Revision (ICD-9) and Tenth Revision (ICD-10) codes, Current Procedural Terminology (CPT), and Healthcare Common Procedure Coding System (HCPCS) codes. A detailed list of our codes and their references are in Supplemental Table 1. Inclusion criteria were similar to those set forth in prior work by Hussan et al. using MarketScan to evaluate the effect of bariatric surgery on colon cancer incidence [59]. Our cohort included adults diagnosed with severe obesity (defined as a BMI ≥ 40 kg/m^2^ or clinically severe obesity) between the years 2012 and 2021, from two databases: (1) patients with private health insurance from contributing employers and (2) supplemental Medicare beneficiaries. MBS cases were classified as adults who underwent elective Roux-En-Y-Gastric Bypass (RYGB) or Vertical Sleeve Gastrectomy (VSG) with documented severe obesity within 1 year prior to surgery [60–63]. The controls were defined as adults with severe obesity and no MBS during the study period. Follow-up was 2012–2021, from the index visit defined as the earliest documentation of severe obesity for the controls or the date of MBS for cases to end date of continuous enrollment.

The event of IBD was defined in two ways: first as the time less than 3 years between the index date and IBD diagnosis date (as defined by outpatient visits and IBD medications) and second as the time 3 years or longer between index date and IBD diagnosis date. The reason for choosing 3 years was because weight gain after MBS tends to happen after 2 years [64], and there can be a delay of up to 1 year in diagnosis of IBD [65].

Patients were excluded if they had any of the following: (i) less than 18 years old; (ii) in the database for < 3 months pre-index visit or < 6 months after index date, (iii) a history of MBS other than VSG or RYGB or MBS with an unknown type or date (iv) other abdominal surgeries prior to MBS (e.g., Nissen fundoplication, small bowel resections, colectomy, transplant, hepatopancreatic surgeries, duodenal switch), (v) a history of an abdominal malignancy (in order to exclude gastric surgeries done for abdominal malignancy), (vi) an organ transplant or (vii) Human Immunodeficiency Virus (HIV) infection [62, 66–75]. To examine the risk of de novo IBD, patients were excluded if they had a history of IBD or use of IBD-related medications prior to index follow up date (Supplemental Fig. 1).

### Definition of IBD in Administrative Data

The primary objective was to investigate the incidence of de novo IBD after MBS, with the hypothesis that patients with obesity who undergo MBS would have the highest incidence of de novo IBD as compared to patients with obesity who did not undergo MBS. Incidence refers strictly to claims-defined incident IBD meeting both diagnostic and medication criteria as defined in the Methods. Specifically, we defined IBD as having the following after the index visit date: (i) two or more visits, one of which was an outpatient visit, with at least one visit for a principal diagnosis of IBD [76, 77], and (ii) the prescription of IBD medications including: 5-ASA (Mesalamine, Sulfasalazine), antimetabolites (6-mercaptopurine, Azathioprine, Methotrexate), biologics (Vedolizumab, infliximab, Adalimumab, Certolizumab, Golimumab, Ustekinumab, Rizankizumab), small molecules (Tofacitinib, Ozanimod), and steroids (prednisone, methylprednisolone, or budesonide).

The secondary objective was assessing IBD severity, hypothesizing that those patients with obesity with de novo IBD after MBS would have a less severe disease course compared to those patients with severe obesity with de novo IBD who did not undergo MBS. Given the limitations of administrative claims data, severity was defined using healthcare utilization–based proxies, including IBD-related medication use (steroids, antimetabolites, biologics/small molecules), IBD-related hospitalizations, and IBD-related surgeries. IBD-related surgeries were defined as strictureplasty, colectomy, enterostomy, anal fistula surgery, incision & drainage, and proctectomy after index visit. IBD-related hospitalizations were defined as hospitalizations with primary diagnosis related to IBD after index date. Objective measures of disease activity such as endoscopic findings, histologic activity, radiographic assessment, or biomarker data were not available in MarketScan and therefore could not be evaluated.

### Definition of Covariates

The demographic covariates included age (in years) and sex (male or female). Charlson comorbidity index (CCI) was calculated using documented comorbidities within 1 year prior to the index visit and categorized as no comorbidities (0); mild (scores of 1–2); moderate (scores of 3–4); or severe comorbidities (scores ≥ 5) [78, 79]. Alcohol or tobacco use was defined by the presence of their respective codes at or prior to index visit and dichotomized as yes or no as done in previous works [59, 80–84].

### Data Analysis

Descriptive statistics such as medians and interquartile ranges (IQRs) were provided for continuous variables while frequencies and percents were provided for categorical variables. MBS and control patients were matched at a ratio of 1:4 by using greedy matching method with a caliper of 0.2 [83]. In this matching analysis, exact matching was done on sex and categorical follow-up time (< 2 years, 2–4 years, 4–6 years, > 6 years) while also including age and each of the 14 individual components of Charlson index (without peptic ulcer disease, metastatic carcinoma, and AIDS/HIV) to calculate the propensity score. This matching algorithm used greedy nearest neighbor method with weights that were dependent on how many control patients were matched to one case patient, such that these weights totaled to a 1:1 ratio (for example, when 4 control patients were matched to 1 case patient, each control was given a weight of 0.25). Standardized differences were calculated on the original (unmatched) as well as on the matched samples.

Cox proportional hazard regressions modeled time from index date to earlier of two dates (diagnosis date with IBD or IBD related outpatient visit) and these models compared MBS to control patients with adjustment for various covariates with the separate models based on two definitions of events (first model with IBD occurring less than 3 years of index date while second model with IBD occurring at 3 years or longer of index date). The first model adjusted for age, sex, combined Charlson comorbidity index, tobacco and alcohol uses while second model adjusted for only sex and tobacco use. The reasons for adjusting for only two covariates in the second model was due to having fewer events compared to first model and hence not overfitting the model was taken into consideration. Chi-square test (or Fisher’s exact test when appropriate) were used to compare MBS to control patients in terms of IBD medications as well as IBD-related surgeries and hospitalizations. All these analyses were performed using SAS version 9.4 (SAS Institute; Cary, NC; www.sas.com). Statistical significance was defined as two-sided alpha < 0.05.

## Results

### Demographics of MBS Versus Control Cohorts

**Fig. 1:**
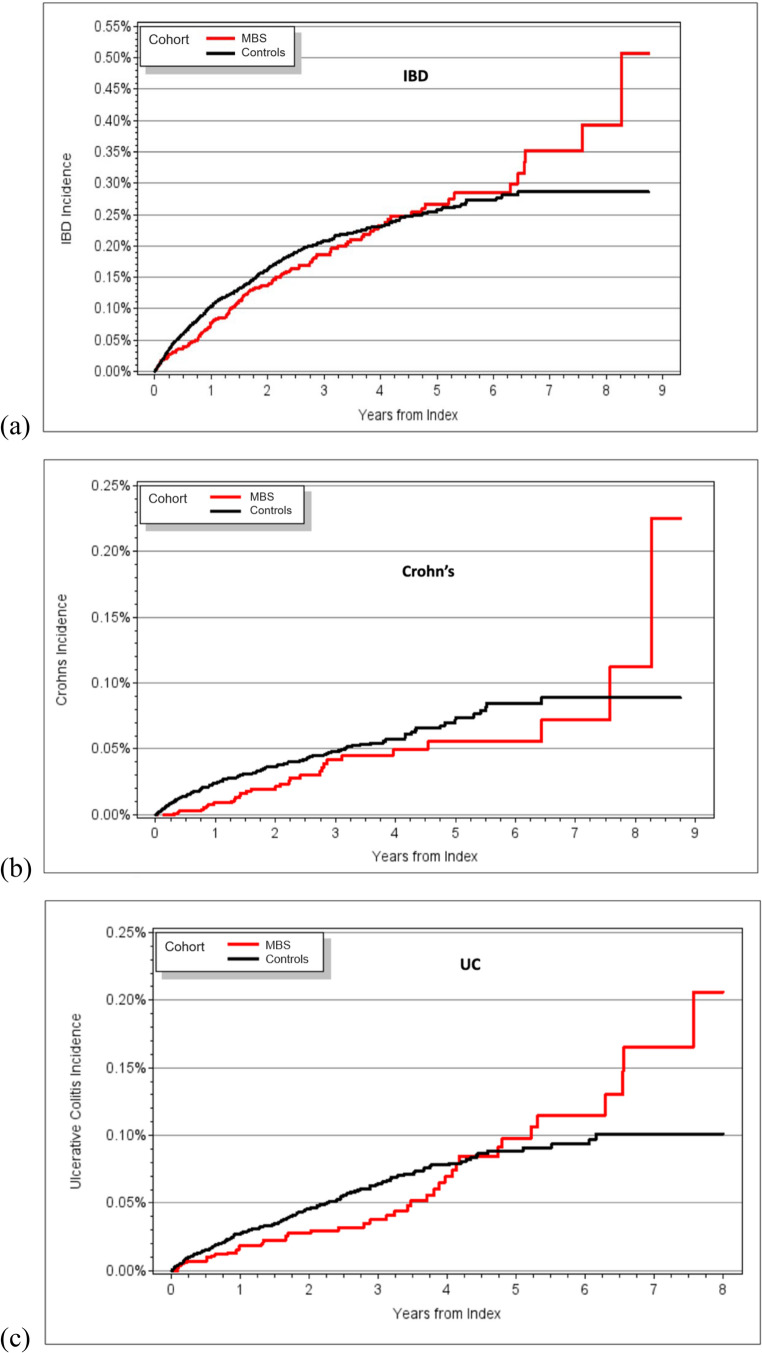
Incidence of IBD in MBS cohort versus controls, stratified by all IBD (**a**), CD (**b**) and UC (**c**)

Our study included 100,832 adults with severe obesity who underwent MBS versus 376,855 propensity-matched controls with severe obesity who never had MBS during the follow-up period. Of those who underwent MBS, 70.3% underwent VSG and the remaining percentage underwent RYGB. The median age was 44 years old, 76.9% were female and median follow-up was 2 years. Our propensity matched cohort had similar distributions of age sex Charlson comorbidity index, and range of follow up between two groups (Table 1). All the standardized differences for the variables included in our propensity score model were smaller than 0.1 in our matched cohort except for mild liver disease, for which the standardized difference was 0.15 which remained greatly reduced in the matched sample compared to original cohort (Supplemental Table 2). The average incidence of IBD was 61.2 per 100,000 individuals per year in the MBS cohort versus 44.4 per 100,000 in the control group (Fig. 1).

**Table 1:** Characteristics of the bariatric cohort and their matched controls

Variable	MBS Patients	Control Patients
Patients included	100,832	376,855
Age, median (Q_1_, Q_3_)	44 (36–52)	44 (36–53)
Female	77,554 (76.9%)	285,349 (75.7%)
Follow-up in years, median (Q_1_, Q_3_) Range	2.0 (1.1–3.6) 0.5–8.8	2.0 (1.0–3.5.0.5) 0.5–8.8
RYGB	29,905 (29.7%)	N/A
VSG	70,927 (70.3%)	N/A
Charlson comorbidity index 0 1–2 3–4 5+	20,883 (20.7%) 48,927 (48.5%) 22,423 (22.2%) 8,599 (8.5%)	83,638 (22.2%) 178,720 (47.4%) 79,678 (21.1%) 34,819 (9.2%)
Tobacco use	13,677 (13.6%)	34,435 (9.1%)
Alcohol use	734 (0.7%)	3,235 (0.9%)
Type 2 diabetes	33,875 (33.6%)	124,202 (33.0%)
IBD rates	158 (0.2%)	622 (0.2%)
Crohn’s disease rates	30 (0.03%)	156 (0.04%)
Ulcerative colitis disease rates	46 (0.05%)	191 (0.05%)
IBD with outpatient incidence rate per 100,000 individual per year*	61.2	44.4
Crohn’s with outpatient incidence rate per 100,000 individual per year*	27.2	13.8
Ulcerative colitis with outpatient incidence rate per 100,000 individual per year*	27.2	16.4

*Derived from the Kaplan Meier curve

### The risk of IBD in the First 3 Years After MBS or Diagnosis of Severe Obesity-Model 1

We stratified the risk of IBD by the time point of before or after 3 years as weight gain after MBS begins after 2 years, and there may be a diagnostic delay in IBD from several months to one year [64, 65]. In the first three years of follow-up, adults with MBS had a 24% lower hazard of developing de novo IBD (adjusted hazard ratio or aHR = 0.76, 95% CI: 0.60–0.95), and a 51% lower hazard of UC than controls (aHR = 0.49, 95% CI: 0.30–0.79, Table 2). The relationship between MBS and control patients in respect to the hazard of CD was not significant (aHR = 0.61, 95% CI: 0.35–1.03, *p* = 0.07). When stratified by MBS type, VSG was associated with significantly reduced IBD hazard, mainly CD, in the first 3 years post-surgery (aHR: 0.46, 95% CI: 0.22–0.96), while RYGB subjects were also at significantly lower hazard of UC (aHR: 0.22, 95% CI: 0.06–0.75, Table 2). This time stratification was prespecified based on prior literature demonstrating weight trajectory changes after MBS and the potential for delayed IBD diagnosis.

**Table 2:** Multivariate analysis of risk of IBD after all MBS, RYGB and VSG stratified by follow-up period

*MBS Type*	*Less than 3 years after index date*^*†*^	*3 years or more after index date*^*†*^
Inflammatory bowel disease		
All MBS vs. Controls	0.76 (0.60–0.95), *p* = 0.02	1.94 (1.00–3.77.00.77), *p* = 0.05
RYGB vs. controls	0.90 (0.62–1.33), *p* = 0.61	1.29 (0.38–4.41), *p* = 0.69
VSG vs. controls	0.69 (0.52–0.92), *p* = 0.01	2.28 (1.02–5.06), *p* = 0.04
**CD**		
All MBS vs. controls	0.61 (0.35–1.03), *p* = 0.07	0.77 (0.30–1.95), *p* = 0.58
RYGB vs. controls	0.87 (0.39–2.00.39.00), *p* = 0.74	0.62 (0.10–3.79), *p* = 0.60
VSG vs. controls	0.46 (0.22–0.96), *p* = 0.04	0.84 (0.28–2.48), *p* = 0.75
**UC**		
All MBS vs. controls	0.49 (0.30–0.79), *p* = 0.004	3.11 (1.32–7.31), *p* = 0.01
RYGB vs. controls	0.22 (0.06–0.75), *p* = 0.02	2.84 (0.65–12.55), *p* = 0.17
VSG vs. controls	0.60 (0.35–1.03), *p* = 0.06	3.22 (1.13–9.19), *p* = 0.03

**†**Index date is defined as surgery date in MBS and earliest diagnosis of severe obesity in controls

### The risk of IBD at 3 Years or More After MBS or Diagnosis of Severe Obesity-Model 2

Adults with VSG had significantly higher hazard for de novo IBD than controls (aHR: 2.28, 95% CI: 1.02–5.06) and borderline significant for adults with all MBS types (aHR = 1.94, 95% CI: 1.00–3.77.00.77, *p* = 0.05). Notably, this higher hazard of IBD was mainly driven by a 3-fold increase in the UC (aHR = 3.11, 95% CI: 1.32–7.31 for all MBS and aHR: 3.22, 95% CI: 1.13–9.19 for VSG). The hazards of CD were insignificant when comparing MBS patients to controls (Table 2).

### IBD Severity in MBS Versus Controls Cohort

There were no significant differences in IBD medication usage between the MBS and controls with IBD, CD or UC (Table 3). Among all IBD patients, 85.4% and 84.2% of MBS and controls patients were prescribed steroids. In contrast, smaller proportions of patients with severe obesity with or without MBS were on biologics/small molecules (5.1% vs. 8.2%) and antimetabolites drugs (8.2% vs. 8.5%). As for IBD-related hospitalizations and surgeries, a significantly higher proportion of MBS patients with UC had colectomies when compared to controls with UC (8.7% vs. 1.6%, *p* = 0.03, Table 4). Other surgeries and hospitalization rates were not significant when compared IBD patients with MBS vs. severe obesity.

**Table 3:** IBD medication use in patients with MBS versus controls

Inflammatory bowel disease	MBS (n = 158)	Controls (*n* = 622)	*p*-value
Biologics/Small Molecules Use Yes No	8 (5.06%) 150 (94.94%)	51 (8.20%) 571 (91.80%)	0.1831
Steroids Use Yes No	135 (85.44%) 23 (14.56%)	524 (84.24%) 98 (15.76%)	0.7102
Antimetabolites Use Yes No	13 (8.23%) 145 (91.77%)	53 (8.52%) 569 (91.48%)	0.9059
Crohn’s disease	**MBS (n=30)**	**Controls (n=156)**	**p-value**
Biologics/Small Molecules Use Yes No	4 (13.33%) 26 (86.67%)	37 (23.72%) 119 (76.28%)	0.2401
Steroids’ Use Yes No	24 (80.00%) 6 (20.00%)	130 (83.33%) 26 (16.67%)	0.6578
Antimetabolites’ Use Yes No	8 (26.67%) 22 (73.33%)	27 (17.31%) 129 (82.69%)	0.2297
Ulcerative colitis	**MBS (n=46)**	**Controls (n=191)**	**p-value**
Biologics/Small Molecules Use Yes No	6 (13.04%) 40 (86.96%)	20 (10.47%) 171 (89.53%)	0.6163
Steroids Use Yes No	32 (69.57%) 14 (30.43%)	138 (72.25%) 53 (27.75%)	0.7165
Antimetabolites Use Yes No	4 (8.70%) 42 (91.30%)	20 (10.47%) 171 (89.53%)	1.0000

**Table 4:** IBD surgeries and hospitalizations in patients with MBS versus controls

*Inflammatory bowel disease*	MBS (*n* = 158)	Controls (*n* = 622)	*p*-value
Strictureplasty Yes No	4 (2.53%) 154 (97.47%)	4 (0.64%) 618 (99.36%)	0.0577
Colectomy Yes No	7 (4.43%) 151 (95.57%)	21 (3.38%) 601 (96.62%)	0.5247
Enterostomy Yes No	3 (1.90%) 155 (98.10%)	3 (0.48%) 619 (99.52%)	0.1013
Anal Fistula Yes No	3 (1.90%) 155 (98.10%)	10 (1.61%) 612 (98.39%)	0.7332
Incision Yes No	2 (1.27%) 156 (98.73%)	3 (0.48%) 619 (99.52%)	0.2677
Proctectomy Yes No	0 (0.00%) 158 (100.00%)	1 (0.16%) 621 (99.84%)	1.0000
# of IBD Surgeries 0 1+	143 (90.51%) 15 (9.49%)	586 (94.21%) 36 (5.79%)	0.0924
# of IBD Hospitalizations 0 1+	74 (46.84%) 84 (53.16%)	239 (38.42%) 383 (61.58%)	**0.0541**
**Crohn’s disease**	**MBS (n=30)**	**Controls (n=156)**	**p-value**
Strictureplasty Yes No	2 (6.67%) 28 (93.33%)	2 (1.28%) 154 (98.72%)	0.1226
Colectomy Yes No	2 (6.67%) 28 (93.33%)	10 (6.41%) 146 (93.59%)	1.0000
Enterostomy Yes No	1 (3.33%) 29 (96.67%)	2 (1.28%) 154 (98.72%)	0.4119
Anal Fistula Yes No	2 (6.67%) 28 (93.33%)	7 (4.49%) 149 (95.51%)	0.6395
Incision Yes No	1 (3.33%) 29 (96.67%)	1 (0.64%) 155 (99.36%)	0.2973
Proctectomy Yes No	0 (0.00%) 30 (100.00%)	1 (0.64%) 155 (99.36%)	1.0000
# of IBD Surgeries 0 1+	24 (80.00%) 6 (20.00%)	137 (87.82%) 19 (12.18%)	0.2501
# of IBD Hospitalizations 0 1+	0 (0.00%) 30 (100.00%)	0 (0.00%) 156 (100.00%)	N/A
**Ulcerative colitis**	**MBS (n=46)**	**Controls (n=191)**	**p-value**
Strictureplasty Yes No	1 (2.17%) 45 (97.83%)	1 (0.52%) 190 (99.48%)	0.3512
Colectomy Yes No	4 (8.70%) 42 (91.30%)	3 (1.57%) 188 (98.43%)	**0.0280**
Enterostomy Yes No	1 (2.17%) 45 (97.83%)	0 (0.00%) 191 (100.00%)	0.1941
Anal Fistula Yes No	0 (0.00%) 46 (100.00%)	4 (2.09%) 187 (97.91%)	1.0000
Incision Yes No	0 (0.00%) 46 (100.00%)	0 (0.00%) 191 (100.00%)	N/A
Proctectomy Yes No	0 (0.00%) 46 (100.00%)	0 (0.00%) 191 (100.00%)	N/A
# of IBD Surgeries 0 1+	41 (89.13%) 5 (10.87%)	183 (95.81%) 8 (4.19%)	0.0740
# of IBD Hospitalizations 0 1+	0 (0.00%) 46 (100.00%)	0 (0.00%) 191 (100.00%)	N/A

## Discussion

The impact of obesity on the risk of IBD is unclear. Examining the effect of weight loss on IBD risk can serve to clarify its role. Evolving literature suggests an increased risk of IBD after MBS, especially after 4 years [27–32, 37, 85]. Therefore, we aimed to further assess the risk and severity of de novo IBD in adults with severe obesity with and without MBS.

We found a temporal relationship between MBS and de novo IBD. Specifically, within three years after MBS, there was a significant reduction in the risk of developing de novo IBD, which was notable for a decrease in Crohn’s disease risk following VSG and ulcerative colitis risk following RYGB. Consistently, using a population based electronic medical record database, Kochhar et al. found a decreased risk of de novo Crohn’s disease after VSG and a decreased risk of de novo ulcerative colitis after RYGB and VSG, predominantly seen in those able to achieve normalization of BMI within 1 year of the MBS [34]. On the other hand, we found an increased risk of de novo IBD and ulcerative colitis after VSG at three years or more after surgery. This is consistent with most other cohort studies that found an increased risk of de novo ulcerative colitis more than 3 years after MBS [35–37]. These data are also consistent with experimental and translational studies identifying an increased risk of colitis after MBS [84, 86, 87].

It should be noted that most prior studies did not stratify de novo IBD risk by MBS type. Most of the MBS types in prior studies were RYGB, while the majority MBS type in our study was VSG. The unique risks associated with VSG versus RYGB may speak to the role of total weight reduction in IBD pathogenesis. While two studies have shown no difference in BMI loss at 5 years between laparoscopic RYGB and VSG [88, 89], in another study, there was greater improvement in total weight loss for RYGB than VSG up to 5 years of follow up [90]. The differences may also speak to the unique impact each MBS type has on the intestinal microbiome and overall metabolic health of the individuals [56, 91]. Still, it cannot be concluded if later diagnosis of de novo IBD is a function of weight regain or the MBS-related microbiome changes, especially since weight loss magnitude could not determined in this dataset [91]. It is also unclear why there were differences in the incidence of CD and UC after MBS. Differences in the microbiome have been shown to exist between patients with VSG and RYGB [59, 92], thus studies should observe the long-term effects of obesity and the microbiome on IBD pathogenesis after MBS, specifically RYGB versus VSG.

There seemed to be no difference in de novo IBD severity in MBS and controls patients as defined by IBD medications. However, around 80% of patients in both the MBS and control cohort were on steroids. Not only could the metabolic effects of steroids contribute to obesity, but it could be a marker of the ineffectiveness of the other medications and relative lack of control of IBD. Pharmacokinetic studies on the biologics and thiopurines have shown high weight is a risk factor for increased drug clearance [93–96]. Braga-Neto found that while most of their patients had mild endoscopic and histologic disease, over time, around 70% of patients required steroids and about 50% required hospitalization due to IBD, despite more patients being on anti-TNF or thiopurines compared to our study [35]. Our study uniquely found that a significantly higher proportion of patients with UC had colectomies comparing MBS patients to the control cohort, which conflicts with another study’s finding of cases having numerically less surgeries than controls, though biologic use was similar between both groups [96]. However, the absolute number of events was small, and claims data do not provide information regarding disease extent, indication for surgery, prior medical optimization, or patient preference. Therefore, this finding should be interpreted cautiously and cannot establish increased intrinsic disease severity. Future studies should include more information on IBD phenotype after MBS, not only through endoscopic, histologic, radiologic and biomarker results but also through details on medication and surgical management. Furthermore, future studies need to investigate biologic pharmacodynamics and pharmacokinetics after weight loss surgery and whether weight loss influences biologics dosing and frequency.

This study is strong as it includes a large cohort of the American population through a national claims database over a 9-year period. The sample size is greater than most studies available and highlights the unique impacts of RYGB and VSG on de novo CD and UC pathogenesis in patients with severe obesity.

Limitations of this study include inability to quantify weight loss after MBS or other elements of the metabolic syndrome such as visceral adiposity. Assessing duration of severe obesity and weight change using ICD codes is difficult and lacks accuracy; the possibility of reverse causality as in IBD causing weight loss would be difficult to isolate in this retrospective database. Even in other retrospective analyses, average BMI did not necessarily normalize after MBS [35–37]. While biologically plausible mechanisms such as weight regain, alterations in bile acid metabolism, and microbiome changes may contribute to these findings, longitudinal weight, metabolic, and microbiome data were not available in this dataset. Longitudinal weight data and measures of weight regain or metabolic parameters were not available, preventing direct assessment of the relationship between weight trajectory and IBD risk. Residual confounding may persist despite propensity matching. Therefore, these interpretations should be considered hypothesis-generating rather than mechanistic conclusions. Future studies need to prospectively compare patients with and without severe obesity seen in a general gastroenterology clinic, aiming to control for the significance of non-IBD abdominal complaints and focus on the impact of duration of obesity and weight change over time in de novo IBD pathogenesis. Assessing annual risk of de novo IBD may help capture the impact of body weight change and visceral adiposity.

Racial, ethnic, and socioeconomic factors that may uniquely influence both obesity and IBD pathogenesis were not able to be captured in this study. This is an American database, therefore the impact of access to care and insurance coverage along with differences in diet and obesity prevalence may not be generalizable to the entire world, though obesity rates are rising worldwide [96–98].

Finally, due to the nature of Marketscan, “incidence” of IBD referred strictly to claims-defined incident IBD meeting both diagnostic and medication criteria as defined in the methods. Furthermore, we were unable to capture the severity of IBD as based on endoscopic, radiologic, histologic and biomarker criteria, as well as pertinent factors related to IBD such as family history and severity of other autoimmune disorders. An astonishingly high number of patients were on steroids in this cohort, which does not necessarily reflect the treatment strategy advocated by American IBD treatment guidelines [99–101]. Future prospective studies are needed to better capture these elements as well as duration of steroid use and contemporaneous biologic use in ways Marketscan cannot.

In conclusion, future groups should seek to prospectively track patients after MBS to analyze the factors that promote sustained weight loss, including changes in their microbiome and inflammatory milieu that may contribute to the risk of developing de-novo IBD. Markers of the metabolic syndrome such as visceral adiposity and comorbidities such as hypertension, hyperlipidemia and diabetes should be accounted for to understand each condition’s impact on de novo IBD and its management. More data on IBD severity including clinical and patient reported outcomes should be collected to understand and treat IBD in this unique set of patients that is rapidly expanding. This information could not only help stratify those patients preparing for MBS who are at risk for IBD and whose gastrointestinal symptoms may need further evaluation before surgery but also help contribute to a better general understanding of IBD pathogenesis. 

## Supplementary Information

Below is the link to the electronic supplementary material.

MOESM 1 (DOCX 92.0 KB)

## Data Availability

This was a retrospective cohort study using the 2012-2021 IBM^®^ MarketScan Research Databases, which contains proprietary de-identified claims data for privately and publicly insured people in the United States [57]. The MarketScan consists of two core claims databases: commercial and Medicare supplement, providing the de-identified healthcare data from over 273 million patients. The MarketScan Research Databases meet the criteria for a limited-use data set and contain none of the data elements prohibited by Health Insurance Portability and Accountability Act of 1996 (HIPAA), thus data is considered deidentified and this project was exempt from Institutional Review Board oversight [58].
