# Supplementary material for: The Impact of Metabolic Bariatric Surgery on Inflammatory Bowel Disease Risk and Outcomes in Adults With Obesity: a Propensity-matched, Nationwide, Analysis
Source: Obes Surg. 2026 May 5;36(6):3078–90. doi: 10.1007/s11695-026-08714-1 (PMC13249756; doi:10.1007/s11695-026-08714-1)
Supplement: Supplementary file 1 — (DOCX 92.0 KB) [file 11695_2026_8714_MOESM1_ESM.docx]

**Supplemental Table 1 Codes used by study inclusion, exclusion, covariates, and outcomes:**

| CODES | | ICD-9 | ICD-10 | CPT | HCPCS |
| --- | --- | --- | --- | --- | --- |
| INCLUSION CRITERIA  {all diagnostic or procedure codes} | | | | | |
| I1. Earliest date of severe obesity (BMI ≥40 kg/m^2^ or clinically severe obesity)  {The earliest date of severe obesity is the index visit for controls} | | Diagnostic codes  278.01  V85.4* | Diagnostic codes  Z68.4*  E66.2  E66.01 |  |  |
| I2. Roux-n-Y gastric bypass (RYGB)  (date of procedure is index visit date)^53, 55-57^ | AND  Must be a non-emergent (elective) encounter | Procedure codes  44.38  44.39  44.31 | Procedure codes  0D16479  0D1647A  0D1647B  0D164J9  0D164JA  0D164JB  0D164K9  0D164KA  0D164KB  0D164Z9  0D164ZA  0D164ZB  0D16079  0D1607A  0D1607B  0D160Z9  0D160ZA  0D160ZB | 43633  43644  43645  43844  43846  43847 | S2085 |
|  | Severe obesity (BMI ≥40 kg/m^2^ or clinically severe obesity)  {at least one code within one year prior to index visit}^57^ | Diagnostic codes  278.01  V85.4* | Diagnostic codes  Z68.4*  E66.2  E66.01 |  |  |
| I3. Vertical sleeve gastrectomy (VSG)  (date of procedure is index visit date)^54, 58^ | Procedure codes, AND  (date of procedure is index visit date)  Must be a non-emergent (elective) encounter | Procedure codes  43.82  43.89 | Procedure codes  0DB64Z3  0DB60Z3 | 43775 |  |
|  | Severe obesity (BMI ≥40 kg/m^2^ or clinically severe obesity)  {at least one code within one year prior to index visit} | Diagnostic codes  278.01  V85.4* | Diagnostic codes  Z68.4*  E66.2  E66.01 |  |  |
| I4. Enrolled at least 3-month PRE-index AND at least 6 months POST-index | | | | | |
| EXCLUSIONS  {all diagnostic or procedure codes} | | | | | |
| E1. Age < 18 | |  |  |  |  |
| E2. GI malignancy  {at or prior to index visit} | | Diagnostic codes  V10.0*  150*-159*  230* | Diagnostic codes  Z85.0*  C15*-26*  D00*  D01* |  |  |
| E3. Inflammatory bowel disease or colitis diagnosis  {at or prior to index visit} | | Diagnostic codes  555*  556*  557*  558* | Diagnostic codes  K50*  K51*  K52*  K55* |  |  |
| E4. Human Immunodeficiency Virus  {at or prior to index visit} | | Diagnostic codes  042*  043*  044*  079.53  279.10  279.19  795.71  V08 | Diagnostic codes  Z21  B20*-24*  R75 |  |  |
| E5. Immunosuppressed status  {at or prior to index visit} | | Diagnostic codes  279.3 | Diagnostic codes  D80*-D84*  D89* |  |  |
| E6. Transplant status  {at or prior to index visit} | | Diagnostic codes  V42.0  V42.1  V42.6  V42.7  V42.8*  V42.9 | Diagnostic codes  Z94.0  Z94.1  Z94.2  Z94.3  Z94.4  Z94.8*  Z94.9 |  |  |
| E7. Other bariatric surgeries (vertical banded gastrectomy, intragastric balloons, lap band, biliopancreatic diversion with duodenal switch)  {During the whole study period for controls, unless if outcome is present after index and prior to E6.  Prior to index visit for MBS} | | Diagnostic codes  V45.86  Procedure Codes  43.7  44.68  44.69  44.93  44.94  44.95  44.96  44.97  44.98  44.99  45.50  45.51  45.90  45.91 | Diagnostic codes  Z98.84  Procedure Codes  0DV6*  0DV7*  0D1* (other than RYGB)  0D190Z9  0DB60ZZ  0DB63Z3  0DB80ZZ  0DP643Z  0DP64CZ  0DQ60ZZ  0DQ64ZZ  0DQ67ZZ  0DQ68ZZ  0DV64CZ  0DW64CZ  0DW643Z | 43659  43770-43774  43842  43843  43845  43848  43886-43888  43850  43855  43860 | S2083  S2082 |
| E8. Colorectal resection  {at or prior to index visit}^59-61^ | | Procedure Codes  48.3*  48.4*  48.5*  48.6*  45.4*  45.7*  45.8*  46.1*  46.2* | Procedure Codes  0DBE*  0DBF*  0DBG*  0DBH*  0DBK*  0DBL*  0DBM*  0DBN*  0DBP*  0DTE*  0DTF*  0DTG*  0DTH*  0DTK*  0DTL*  0DTM*  0DTN*  0DTP* | 44139-44160  44204-44227  45395  45397  45110  45111  45112  45113  45114  45116  45119  45120  45126  45160  45171  45172  45190  44204  44205  44206  44207  44208  44210  44211  44212 |  |
| E9. Other abdominal surgeries  {at or prior to index visit for controls. Prior to index for cases}  Includes esophageal resection, gastric resection, fundoplication, hepatic resections, cholecystectomy, pancreatectomy, small bowel resection, bypass to colon or cutaneous, hepatobiliary operations, small bowel resections and transplant surgeries^57,62-68^ | | Procedure Codes  42.4*  43.5*  43.6*  43.81  43.9*  44.32  44.5  44.6*  44.9*  45.6*  50.2*  50.3  50.4  50.5*  51.2*  52.5*  52.6  52.7  52.8* | Procedure Codes  0D1* (other than RYGB)  0D5*  0DB*  (other than VSG)  0DT*  0DV*  0DW*  0DX*  0DY*  0F1*  0F5*  0FB*  0FT*  0FV*  0FW*  0FY* | 43100-43135  32665  43279-43289  43324 43325 43326 43327 43328  43279  43330  43331  43605-43652  43999  44120  44121  44125 44130 44200  44203  45136  47120-47130  47133-47147  47562-47564  47600-47620  56340-56342  49310  48105-48160  48550-48556  49310  49311  47562 |  |
| E10. Gastric outlet obstruction  {on index visit or within 1 year prior to index visit} | | Diagnostic codes  537.0 | Diagnostic codes  K31.1 |  |  |
| E11. Gastric ulcers  {on index visit or within 1 year prior to index visit} | | Diagnostic codes  531*-533* | Diagnostic codes  K25*-K27* |  |  |
| E12. Use of IBD medications  {at or prior to index visit}   - - 5-ASA     - Mesalamine     - Sulfasalazine   - Antimetabolite     - 6-mercaptopurine     - Azathioprine     - Methotrexate   - Biologics     - Vedolizumab     - Infliximab     - Adalimumab     - Certolizumab     - Golimumab     - Ustekinumab     - Rizankizumab   - Small molecules     - Tofacitinib     - Ozanimod | |  |  |  |  |
| VARIABLES (All Diagnostic codes) | | | | | |
| V1. Tobacco smoking status  {at or prior to index visit}^73, 75^ | | Diagnostic codes  305.1  V15.82 | Diagnostic codes  F17*  T65.2  Z71.6  Z72.0  Z87.891 |  |  |
| V2. Alcohol use  {at or prior to index visit}^74^ | | Diagnostic codes  291*  305.0  303  357.5  425.5  535.30  535.31  571.1  571.2  571.3  E860.0 | Diagnostic codes  F10*  Z71.4  G31.2  Y90*  R78.0  T51.0*  T51.9*  K29.2*  I42.6 |  |  |
| V3. Charlson comorbidity index {on index visit or within 1 year prior to index visit}^71^ | |  |  |  |  |
| V4. Type II Diabetes Mellitus  {on index visit or within 1 year prior to index visit}^72^ | | Diagnostic codes  250* | Diagnostic codes  E11* |  |  |
| OUTCOMES | | | | | |
| O1. IBD (Only include patients with two codes, with at least one DX1 or PDX. The second one can be any DX). The O1 date will be the earliest date of either codes.  If have both O1a and O1b, then would choose the last two encounter diagnosis since the IBD diagnosis can change with time.^69,70^ | | Diagnostic codes  555*  556*  558.9 | Diagnostic codes  K50*  K52.3 |  |  |
| O1a Crohn’s (Only include patients with two codes, with at least one DX1 or PDX. The second one can be any DX). The O1a date will be the earliest date of either codes. | | Diagnostic codes  555* | Diagnostic codes  K50* |  |  |
| O1b Ulcerative colitis (Only include patients with two codes, with at least one DX1 or PDX. The second one can be any DX). The O1b date will be the earliest date of either codes. | | Diagnostic codes  556* | Diagnostic codes  K51* |  |  |
| IBD Medication use within one month prior to O1 till end of follow up (yes/no)   - - 5-ASA     - Mesalamine     - Sulfasalazine   - Antimetabolite     - 6-mercaptopurine     - Azathioprine     - Methotrexate   - Biologics     - Vedolizumab     - Infliximab     - Adalimumab     - Certolizumab     - Golimumab     - Ustekinumab     - Rizankizumab   - Small molecules     - Tofacitinib     - Ozanimod   - Steroids     - Prednisone     - Methylprednisolone     - Budesonide | |  |  |  |  |

**Supplementary Table 2 Standardized differences comparing original to matched sample**

| **Variable** | **Standardized difference (original unmatched sample)** | **Standardized difference (matched sample)** |
| --- | --- | --- |
| Age | 0.261 | 0.047 |
| Sex | 0.329 | 0.028 |
| Follow-up time (categorized) | 0.149 | 0.020 |
| MI | 0.041 | 0.002 |
| CHF | 0.060 | 0.001 |
| PVD | 0.026 | 0.005 |
| Cerebrovascular Disease | 0.055 | 0.000 |
| Dementia | 0.045 | 0.010 |
| Chronic Pulmonary Disease | 0.163 | 0.007 |
| Connective Tissue Disease-Rheumatic Disease | 0.020 | 0.002 |
| Mild Liver Disease | 0.512 | 0.151 |
| Diabetes without complications | 0.187 | 0.008 |
| Diabetes with complications | 0.029 | 0.005 |
| Paraplegia and Hemiplegia | 0.041 | 0.012 |
| Renal Disease | 0.065 | 0.007 |
| Cancer except malignant neoplasm of skin | 0.059 | 0.013 |
| Moderate or Severe Liver Disease | 0.003 | 0.008 |

**Supplemental Figure 1. Study exclusion and inclusion criteria**

Exclusion criteria applied (n=844,248):

- Age < 18 years old
- Gastrointestinal malignancy
- Inflammatory bowel disease or colitis diagnosis
- Human immunodeficiency virus
- Immunosuppressed status
- Transplant status
- Other bariatric surgeries
- Colorectal resection
- Other abdominal surgeries
- Gastric outlet obstruction
- Gastric ulcers
- Use of IBD medications at or prior to index visit

2,730,092 patients identified

**Identification**

Two components of Charlson Comorbidity Index applied for exclusion, peptic ulcer disease and metastatic carcinoma (n=6,816):

- 6,529 had only metastatic carcinoma
- 286 had only peptic ulcer disease
- 1 patient had both

1,885,844 patients remained

**Screening**

1,879,028 patients remained for analysis

100,832 adults with severe obesity who underwent MBS

376,855 propensity-matched controls with severe obesity adults who did not have MBS controls with severe obesity surgery versus 376,855 propensity-matched controls with severe obesity who underwent MBS versus 376,855 propensity-matched controls with severe obesity

**Included based on propensity scored matching**
